# CRISPR-Based Isothermal Next-Generation Diagnostic Method for Virus Detection in Sugarbeet

**DOI:** 10.3389/fmicb.2021.679994

**Published:** 2021-07-08

**Authors:** Vanitharani Ramachandran, John J. Weiland, Melvin D. Bolton

**Affiliations:** United States Department of Agriculture, Agricultural Research Service, Northern Crop Science Laboratory, Fargo, ND, United States

**Keywords:** RT-RPA, CRISPR, virus, rhizomania, BNYVV, sugarbeet

## Abstract

Rhizomania is a disease of sugarbeet caused by beet necrotic yellow vein virus (BNYVV) that significantly affects sugarbeet yield globally. Accurate and sensitive detection methods for BNYVV in plants and field soil are necessary for growers to make informed decisions on variety selection to manage this disease. A recently developed CRISPR-Cas-based detection method has proven highly sensitive and accurate in human virus diagnostics. Here, we report the development of a CRISPR-Cas12a-based method for detecting BNYVV in the roots of sugarbeet. A critical aspect of this technique is the identification of conditions for isothermal amplification of viral fragments. Toward this end, we have developed a reverse transcription (RT) recombinase polymerase amplification (RPA) for detecting BNYVV in sugarbeet roots. The RT-RPA product was visualized, and its sequence was confirmed. Subsequently, we designed and validated the cutting efficiency of guide RNA targeting BNYVV via *in vitro* activity assay in the presence of Cas12a. The sensitivity of CRISPR-Cas12a *trans* reporter-based detection for BNYVV was determined using a serially diluted synthetic BNYVV target sequence. Further, we have validated the developed CRISPR-Cas12a assay for detecting BNYVV in the root-tissue of sugarbeet bait plants reared in BNYVV-infested field soil. The results revealed that BNYVV detection is highly sensitive and specific to the infected roots relative to healthy control roots as measured quantitatively through the reporter signal. To our knowledge, this is the first report establishing isothermal RT-RPA- and CRISPR-based methods for virus diagnostic approaches for detecting BNYVV from rhizomania diseased sugarbeet roots.

## Introduction

Plant viruses are serious pathogens significantly affecting agriculture productivity and economy in a wide variety of crops around the world (Loebenstein, 2008). Sugarbeet (*Beta vulgaris*) cultivated for its root is a major source of sucrose for natural sweetening worldwide. Many different viruses infect sugarbeet, and among those, beet necrotic yellow vein virus (BNYVV), which causes the devastating rhizomania disease, is of global importance, affecting yield loss through reduced root mass and sugar content and increased impurities during sucrose extraction (Rush et al., 2006). BNYVV, the main causal pathogen of rhizomania, is a multipartite RNA viral member of the genus *Benyvirus* belonging to the Benyviridae family (Tamada and Baba, 1973; Gilmer and Ratti, 2017). The virus is transmitted through the soil into susceptible plants by *Polymyxa betae*, an obligate pathogen of sugarbeet (Fujisawa and Sugimoto, 1977). Rhizomania was discovered in Italy in the 1950s and later reported from sugarbeet-growing regions around the world. The first report of the disease in the Western Hemisphere was in the United States (Duffus et al., 1984), and since then, it has been found in various locations ranging from Brazil to Canada and including the Northern Plains (Wisler et al., 1997; Weiland et al., 2019), the largest sugarbeet-producing region in the United States (Bangsund et al., 2012).

Rhizomania disease management measures principally rely on resistance genes bred into commercial varieties specifically developed against BNYVV (Rush et al., 2006). Hence, accurate and sensitive detection of BNYVV in plants and infected fields soils is crucial in appropriating management strategies that include varietal selection, non-host crop rotations, and evaluating resistance levels of sugarbeet breeding lines. Enzyme-linked immunosorbent assay (ELISA), a protein-based detection technology, has been used for many years for field soil evaluations because of the ease of implementation and availability of reagents commercially (Torrance et al., 1988). Later, molecular biology-based standard reverse transcription (RT)-PCR and nested RT-PCR methods were developed to increase the sensitivity of BNYVV detection (Henry et al., 1995; Morris et al., 2001). An immunocapture loop-mediated isothermal amplification (IC-RT-LAMP) method subsequently was reported to be more sensitive (Almasi and Almasi, 2017). Hitherto, it has been a challenge developing an ideal assay that can be simple to perform, sensitive, specific, rapid, and at the same time cost-effective and tractable to high-throughput diagnostics.

Since the recent discovery of collateral activity of clustered regularly interspaced short palindromic repeats (CRISPR)-associated (Cas) proteins (Chen et al., 2018), technologies employing the phenomenon have advanced tremendously from the modification of genomes through the development of novel diagnostic capabilities, including those for viruses. Of the Cas proteins, Cas12a is an RNA-guided DNA endonuclease that, upon mediating target double-stranded (ds) DNA cleavage, acquires an indiscriminate single-stranded (ss) DNA cleavage activity. This collateral activity has been exploited for developing sensitive, specific, and rapid detection methods for human viruses including human papilloma (HPV) viruses (Chen et al., 2018), as well as Dengue and Zika viruses (Curti et al., 2020). In plants, CRISPR-Cas system-based virus detection is in the early stages of development. Recently, CRISPR-Cas12a-based assay has been developed for detecting potato virus X (PVX), potato virus Y (PVY), and tobacco mosaic virus (TMV) in the leaf tissues of the model plant *Nicotiana benthamiana* (Aman et al., 2020), as well as a slightly modified method for detecting viruses in apple leaf samples (Jiao et al., 2020).

In this study, we report a new molecular diagnostic method based on the CRISPR-Cas12a system termed DETECTR (DNA Endonuclease Targeted CRISPR Trans Reporter) technology (Chen et al., 2018) for detecting BNYVV in the roots of sugarbeet. Template DNA amplification of viral fragments under isothermal conditions is crucial for developing CRISPR-Cas12a-based diagnostics method. We have developed an inexpensive isothermal one-step RT recombinase polymerase amplification (RPA) method and confirmed the sequence identity of the RT-RPA amplicon representing the BNYVV sequence. Further, the CRISPR-based BNYVV detection method was evaluated, and the sensitivity was determined in the roots of sugarbeet baited for rhizomania using field soils.

## Materials and Methods

### Plant Growth and Virus Recovery From Soil Samples

Soil samples were obtained from the sugarbeet production areas of North Dakota and Minnesota courtesy of agriculturists from the Southern Minnesota Beet Sugar Cooperative (Renville, MN, United States). Sugarbeet seed susceptible variety HD0484 was used for this study (SESVanderhave, Fargo, ND, United States). For healthy control, susceptible sugarbeet seeds were planted into Sunshine Mix with sand of 1:1 ratio (Sungro Horticulture, Agawam, MA, United States). Slow-release fertilizer (Multicote; Sungro Horticulture, Agawam, MA, United States) was added following the manufacturer’s instructions. Plants were grown in a greenhouse under standardized conditions at 24°C/18°C day/night with 8 h of supplemental light per day, and water was added directly as needed. Six weeks after planting in infested soil, plants were harvested, and a root sample consisting of two to three plants was taken from each pot. Roots were washed gently in a tray containing water, taking care to retain fine root hairs, damp dried on paper towel, and stored at -80°C until used for RNA extraction.

### Reverse Transcription-Recombinase Polymerase Amplification

RPA reactions were performed using TwistAmp liquid basic kit (TwistDx, Cambridge, United Kingdom). Primers were designed and synthesized from Integrated DNA Technologies (IDT; Coralville, IA, United States) for amplifying a 465-base pair (bp) fragment of BNYVV RNA-1 (Table 1). The RPA reactions were setup as follows. Plasmid DNA carrying synthetic genomic sequences of BNYVV RNA-1 (Weiland et al., 2020) was diluted to test the primer efficiency on RPA. The RPA reaction was set up with the following reagents: 2 μl of template, 2.5 μl each of forward (VR-1, 32 nts) and reverse (VR-2, 33 nts) primers (10 μM), 2 × reaction buffer 25 μl, dNTPs 4 μl (10 mM), 10 × basic E-mix 5 μl, 20 × core reaction mix 2.5 μl, and MgOAc 2.5 μl, and remaining volume adjusted with nuclease-free water to a final volume of 50 μl. The reaction was incubated at 40°C for 30 min, and RPA amplicons were analyzed by agarose gel electrophoresis. For the RT-RPA-based amplification of BNYVV from sugarbeet root tissue, total RNA from healthy and rhizomania baited roots were used. One hundred milligrams of cleaned root tissue was ground using a pulverizer (SPEX, Fisher Scientific, MA, United States), and then total RNA was extracted using RNeasy Plant mini kit (Qiagen, Germantown, MD, United States), with final RNA concentration determined using a Nanodrop (Thermo Fisher Scientific, Waltham, MA, United States). Equal concentrations of total RNA from healthy and rhizomania-infected roots were used for setting up RT-RPA reactions. RT-RPA reactions were set up using total RNA (100 ng) for rhizomania baited roots and healthy roots separately in a 50-μl reaction containing forward (VR-1) and reverse (VR-2) primers each 2.5 μl (10 μM), 2 × reaction buffer 25 μl, dNTPs 4 μl (10 mM), 10 × basic E-mix 5 μl, 20 × core reaction mix 2.5 μl (TwistDx, Cambridge, United Kingdom), and 2 μl of M-MuLV Reverse Transcriptase (NEB, Ipswich, MA, United States), and MgOAc 2.5 μl, and the remaining volume was adjusted with nuclease-free water. After gently mixing and collecting the contents of the tubes with a brief spin, the reactions were incubated at 42°C for 60 min. To visualize the RT-RPA products, an aliquot of the RPA reaction was transferred to a new tube, and to that, EDTA was added to a final concentration of 20 mM and held at room temperature for 5 min. After a brief spin, the contents were loaded onto the agarose gel containing SyberSafe stain (Invitrogen, Waltham, MA, United States). Following electrophoresis, DNA products were visualized using ChemiDoc (Bio-Rad, Hercules, CA, United States). Gel elution of the RT-RPA product was carried out using a Gel extraction kit (Qiagen, Germantown, MD, United States) and subjected to Sanger sequencing (MCLAB, San Francisco, CA, United States).

**TABLE 1:** Sequences of primers, ssDNA reporter, CRISPR-guide RNA, and target used in this study.

Name	Sequence
VR-1	5′-gcgttctgattatcagaatcaacgagttggtg-3′ (3064–3095)
VR-2	5′-atatgttcaccagtctcatcggaataatgaatg-3′ (3528–3496)
VR-3	5′-cgttctgattatcagaatcaac-3′ (3065–3086)
VR-4	5′-atatgttcaccagtctcatcg-3′ (3528–3508)
Reporter	/56-FAM/TTATT/BHQ-1
BNY-gR1	5′-UAAUUUCUACUAAGUGUAGAUCAGCCUAUG AUUGGCGGUUGC-3′ (3180–3200)
BNYVV synthetic template DNA (GenBank accession number MT227164)	gcgttctgattatcagaatcaacgagttggtgatgagcttctttcttggga ctttcacacgcctcacaaagcattggatgttactggtaagcaaattatttt tgttgatgagtttacagcctatgattggcggttgctagctgtgttggcttata gaaatcatgcccatactatttacttagttggtgatgagcagcagactggt attcaagagggtcgtggagaaggaatatcgatacttaacaaaattgat ctgtctaaggtttctacacatgttccaatcatgaactttagaaatcctgtcc gtgatgttaaggtattaaattatctgttcgggtctcgtatggttcctatgtct tccgttgaaaagggatttagtttcggggatattaaagaattttcgtctttgtc aaatatcccagacactaaaatcattcattattccgatgagactggtgaa catat (3064–3528)

**Coordinates relative to target BNYVV RNA-1 are indicated in parenthesis. Underlined sequence refers to guide RNA of BNYVV RNA-1.**

### CRISPR Guide RNA Validation Assay

The DNA fragment representing BNYVV RNA-1 (GenBank accession number MT227164) genomic region comprising 465 bp chosen as the target for CRISPR-assay was synthesized (Table 1) and obtained in a standard cloning vector (Genewiz, South Plainfield, NJ, United States). Within the synthetic fragment, a PAM sequence for Cas12a was identified, and a guide RNA of 21 nts targeting the chosen fragment of BNYVV was designed and synthesized (IDT, Coralville, IA, United States). Cas12a from *Lachnospinaceae bacterium* used in guide RNA validation was purchased from New England Biolabs (NEB, Ipswich, MA, United States). For testing the specific cleavage of target DNA in an *in vitro* biochemical assay, a linear dsDNA template (464 bp) was generated via PCR using primers VR-3 and VR-4 and synthetically cloned plasmid DNA containing the selected BNYVV sequence (Table 1). First, the ribonucleoprotein complex was preassembled by using 30 nM Cas12a and 40 nM guide RNA in the buffer supplied with Cas12a and incubated at 25°C for 10 min. The *in vitro* cleavage reaction was setup by addition of 10 μl of preassembled Cas12a (30 nM) and guide RNA (40 nM) complex to the template (20 nM) along with 1 × concentration of the cognate buffer in a volume of 30 μl where final volume was made up with nuclease-free water and incubated at 37°C for various time points from 5, 10, 20, and 30 min to determine the cleavage kinetics over time. The cleavage reactions were analyzed on agarose gel and photographed using ChemiDoc (Bio-Rad, Hercules, CA, United States).

### CRISPR-Cas12a Reporter Assay

For CRISPR-based reporter assay, the ribonucleoprotein complex was preassembled using Cas12a (30 nM), guide RNA (40 nM), and ssDNA reporter (30 nM) in 1 × concentration of Cas12a cognate buffer at 25°C for 10 min. The ssDNA reporter with fluorophores 5’6-carboxyfluorescein (FAM) and 3’ Black Hole Quencher-1 (BHQ-1) as indicated in Table 1 was synthesized (IDT, Coralville, IA, United States). To evaluate the sensitivity of the developed CRISPR-based assay for detecting BNYVV, a linear dsDNA PCR fragment produced from synthetic BNYVV was used. Tenfold dilutions were made, and 2 μl from each dilution, which is equivalent to 10, 1, 0.1, and 0.01 pM, was used in CRISPR-reporter assay in a volume of 40 μl in a 96-well plate (Bio-Rad, Hercules, CA, United States). The reactions were incubated at 37°C for 60 min, and output fluorescence signal was measured at 485-nm excitation and 535-nm emission in a Tecan Spark Ultra plate reader (TECAN, Mannedorf, Switzerland). For BNYVV detection from sugarbeet root samples, first, RT-RPA was conducted using 100 ng of total RNA extracted from sugarbeet root tissue as described in this section in a volume of 50 μl using the primers VR-1 and VR-2. The RT-RPA reactions were diluted up to 10^–5^, and from each dilution, 5 μl was used for the CRISPR-assay. For CRISPR assay background noise alleviation, the no template control (NTC) reaction was included in the assay, which was subtracted from test samples prior to plotting the graphs.

## Results

### Isothermal Amplification and Sequence Confirmation of BNYVV From *B. vulgaris* Root Using RT-RPA

**FIGURE 1:**
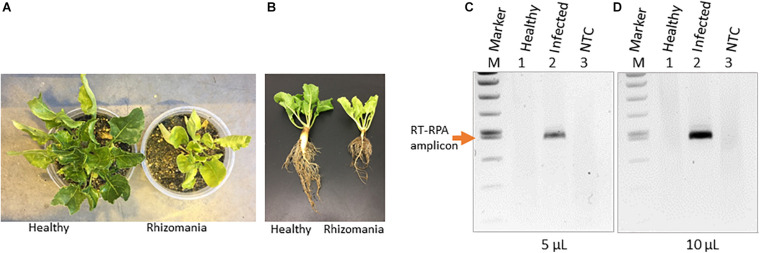
Rhizomania baiting and RT-RPA-mediated detection of BNYVV from *B. vulgaris*. **(A)** Whole-plant pictures of *B. vulgaris* baited for rhizomania under greenhouse condition in pots using infected field soil 6 weeks post inoculation. **(B)** Representative individual plants showing root phenotype associated with rhizomania. Extreme care was taken to gently remove the soil from root. **(C,D)** Detection of RT-RPA amplicon on agarose gel. Even 5 μl **(C)** and 10 μl **(D)** loading volumes show strong visual band from a 50-μl reaction. Lanes: Infected refers to sugarbeet roots baited for rhizomania disease from field soil. Healthy refers to root obtained from sugarbeet grown in potting mix used for growing plants under laboratory conditions. NTC stands for no template control, and M stands for size marker.

Rhizomania disease baiting in sugarbeet roots was accomplished by growing plants using rhizomania-infested soil. The presence of rhizomania in the soil investigated in this study was confirmed by ELISA testing for BNYVV presence (data not shown). Under greenhouse conditions, sugarbeet grown in pot containing soil obtained from field showed yellowish foliar phenotype, while in healthy plants grown in potting mix (no field soil), the leaves remained noticeably more-green (Figure 1A). The representative root phenotypes of rhizomania-infected versus healthy sugarbeet plantlets are shown in Figure 1B. The root of healthy plants root was large with dense rootlets, whereas the rhizomania diseased root was thinner with less-dense rootlets (Figure 1B).

As a prelude to the development of a CRISPR assay, it is essential to establish isothermal amplification of target templates, and for that, we decided to utilize an RPA-based method. In our case, BNYVV being an RNA virus, we have developed an RT-RPA method directed at its viral genome to achieve template amplification under isothermal condition. To accomplish RT-RPA of BNYVV, we designed primers that amplified a 465-bp fragment of BNYVV RNA-1 in a single tube using reagents from the TwistAmp liquid basic kit along with M-MuLV reverse transcriptase from total RNA extracted from sugarbeet root samples. In an effort to visualize the RT-RPA products on the gel, we discovered that addition of EDTA to a final concentration of 20 mM to the RT-RPA reaction facilitated the observation of a sharp band readily detectable on agarose gel upon loading even 5 and 10 μl out of the total 50-μl reaction volume (Figures 1C,D). The RT-RPA analysis of total RNA isolated from sugarbeet roots baited for rhizomania from infected field soil revealed the production of an amplification product of a 465-bp fragment as expected (Figures 1C,D). No such amplification product was obtained in the root samples from healthy sugarbeet controls. Absence of amplified product with NTC revealed the specificity of reagents used in the RPA assays. Next, to confirm the sequence authenticity, the RT-RPA-amplified fragment was gel purified, and Sanger sequencing analysis revealed the amplicon indeed had the expected BNYVV RNA-1 sequence. Taken together, we have developed an isothermal one-step RT-RPA assay to amplify BNYVV from sugarbeet roots baited for rhizomania from field soil, optimized conditions to visualize the RT-RPA products on gel, and confirmed the identity of the underlying sequence useful for downstream molecular analysis.

### Validation of Guide RNA Designed to Targeting BNYVV Genome

**FIGURE 2:**
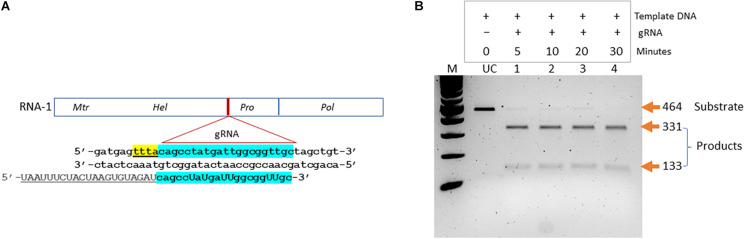
Validation of designed guide (g)RNA targeting BNYVV genome. **(A)** Diagrammatic representation of BNYVV RNA1 genome showing the location of the guide RNA (gRNA) target site and the corresponding sequence information. Sequence keys: gRNA, blue highlight; CRISPR-RNA, underlined; and PAM, yellow highlight. **(B)** *In vitro* activity assay. Substrate (464 bp), linear dsDNA fragments comprising the target sequence are assayed along with the guide RNA and resolved on agarose gel electrophoresis. Products refer to cleaved DNA fragments (331 and 133 bp). Lanes 1–4, time course analysis in 5, 10, 20, and 30 min indicated at the top of the gel image. UC refers to uncut template DNA.

We aimed at developing a CRISPR-based diagnostics assay for BNYVV detection in sugarbeet. In the CRISPR-Cas system-based detection methods, guide RNAs play key roles in directing target-specific cleavage upon which Cas enzyme attains the novel non-specific nucleolytic activity. Therefore, it is important to validate the cleavage potential of designed guide RNA before being used in assay development. We have designed a guide RNA (BNY-gR1) targeting the RNA-1 of BNYVV (Table 1). In the BNYVV genome, RNA-1 is important by encoding enzymes essential for virus replication. Figure 2A shows a diagrammatic representation of the location of the designed guide RNA on the RNA-1 of BNYVV. To evaluate Cas12a-meadiated *cis* cleavage efficiency of the designed guide RNA, we conducted an *in vitro* activity assay by incubating the PCR products derived from the synthetic BNYVV RNA-1 along with the Cas12a enzyme at different time points 5, 10, 20, and 30 min at 37°C and analyzed on agarose gel electrophoresis. As expected, BNY-gR1 cleaved the 464-bp linear template DNA releasing into two fragments of 331 and 133 bp, and time course analysis revealed that the efficiency of cleavage activity was almost completed within 5 min of incubation, indicating that the designed guide RNA is highly efficient in cleaving its target DNA (Figure 2B). The demonstrated *in vitro* activity assay fulfills the prerequisite validation of the cleavage potential of BNY-gR1 through its ability to form an RNA–protein complex with Cas12a and recognizing the PAM sequence on the template DNA, thus catalyzing sequence-specific cleavage releasing the expected products (Figure 2B).

### Evaluation of Sensitivity of CRISPR-Cas12a-Based Detection of BNYVV Using Synthetic Targets

**FIGURE 3:**
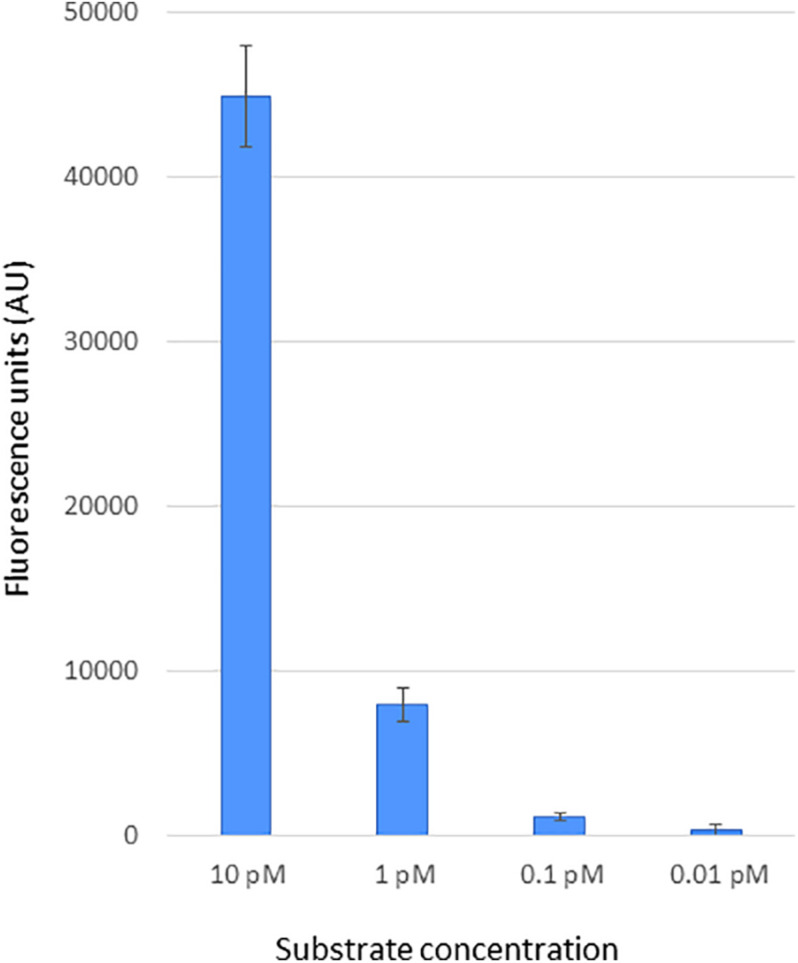
Sensitivity of CRISPR-Cas12a-based BNYVV detection using synthetic DNA targets. The BNYVV RNA1 template DNA encompassing the CRISPR RNA target used in this assay was obtained via PCR using a synthetic DNA. To determine the detection limit, the target DNA fragment was serially diluted 10-fold and subjected to CRISPR-Cas12a reporter assay as mentioned in the Materials and methods section. The output signal of the assay reactions was quantified using a plate reader. In this assay, reactions with no template served as the negative control and were used for background subtraction. The *y*-axis represents background-subtracted values of fluorescence units, and *x*-axis shows dilutions of target DNA indicated in picomolar (pM). Error bars, standard deviation (STDEV) on replicates (*n* = 3).

To assess the sensitivity of CRISPR-Cas12a-based DETECTR assay for BNYVV detection, we used a linear PCR amplicon (464 bp) generated from a synthetic DNA template that has BNYVV RNA-1 genomic viral sequences encompassing the PAM and gRNA target sequences. Ten-fold serially diluted template DNA of BNYVV was used to evaluate sensitivity of the CRISPR-Cas12 reporter assay as described in “Materials and Methods” section, and the results revealed that fluorescence signal was obvious in the reactions that had BNYVV template DNA compared to signal generated for no template negative controls (Figure 3). Further, a linear trend of signal reduction correlating with decreasing amounts of template DNA concentration, and with a detection limit of 0.1 pM, template DNA was observed (Figure 3). We noticed that when template concentration was over 10 pM, the fluorescence signal was saturated (data not shown). These results showed that preassembled Cas12a-guide RNA complex specifically cleaved the linear target dsDNA, and as a consequence, the Cas12a acquired a collateral non-specific ssDNA cleavage function, which in turn cleaved *in trans* the fluorescently labeled ssDNA reporter releasing the fluorophore emitting a measurable output fluorescence signal (Figure 3). Altogether, we have developed a CRISPR-Cas12a-based method for BNYVV detection and determined the sensitivity of the method capable of detection at 0.1 pM concentration based on synthetic DNA target of BNYVV.

### CRISPR-Cas12a-Based Detection of BNYVV in *B. vulgaris* Roots Baited for Rhizomania From Field Soil

**FIGURE 4:**
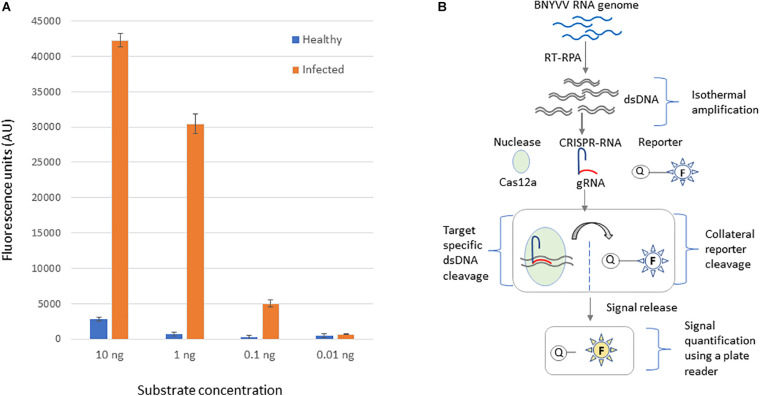
CRISPR-Cas12-based detection of BNYVV in root tissue of *B. vulgaris* baited for rhizomania using infected field soil. **(A)** The template DNA used in this assay was obtained through RT-RPA from rhizomania-infected and healthy roots of sugarbeet. To determine the limit of detection, the RT-RPA product was diluted and subjected to CRISPR-Cas12a reporter assay as described in the Materials and methods section. No template reaction serves as the negative control and was used for background subtraction. Values plotted on the *y*-axis represent background-subtracted fluorescence, and *x*-axis represents dilution concentrations of the RT-RPA-generated template DNA. Error bars represents standard deviation (STDEV) on replicates (*n* = 3). **(B)** Schematic illustration of step-by-step processes involved in the development of CRISPR-Cas12a-based BNYVV diagnostics for rhizomania in sugarbeet roots.

Next, we investigated application of the developed CRISPR-Cas12a-based virus diagnostic assay for detecting BNYVV in the roots, the most highly impacted organ due to rhizomania. Rhizomania-infected roots were obtained by baiting for the disease from field soil, and as a control, healthy roots grown on non-field soil. First, RT-RPA was performed at isothermal conditions using total RNA isolated from rhizomania baited roots and healthy roots along with an NTC. To determine sensitivity of the CRISPR-Cas12a-based assay, these RT-RPA reactions were serially diluted 10-fold, and 5 μl from dilution used in the CRISPR-Cas12a reaction that contains fluorescently labeled ssDNA reporter. After incubating at 37°C for 60 min, the results revealed dramatic strong fluorescence signal in the reactions that had RT-RPA template from rhizomania-infected roots compared to the signal obtained for reactions with healthy root samples (Figure 4A). Signal observed with NTC was considered background, and this value was subtracted from the signals that were obtained for rhizomania-containing roots and healthy root samples to alleviate background noise accompanying the reagents. A linear correlation of signal reduction with increasing levels of serial dilution was observed, and limit of sensitivity was 0.1-ng concentration (Figure 4A). Of note, the field soil tested here showed positive results for rhizomania in a different experiment using ELISA (data not shown). Lastly, we present, a diagrammatic illustration of the various stages of the CRISPR-Cas12a-based method development that were achieved for BNYVV detection, which involves Cas12a enzyme, CRISPR-guide RNA, and ssDNA reporter and how the information is transduced from viral RNA into a detectable signal (Figure 4B). In summary, we have developed an isothermal RT-RPA-based CRISPR-Cas12a diagnostic method to detect BNYVV in rhizomania-infected roots of sugarbeet.

## Discussion

Rhizomania caused by BNYVV is a disease of global importance because it affects sugarbeet yield and productivity significantly. Accurate and sensitive diagnostic methods are essential for formulating strategies for disease management. This study presents the development of the CRISPR-Cas12a-based method for BNYVV detection in rhizomania roots. Isothermal amplification of virus-derived template is an essential component of CRISPR-based detection scheme. We have developed a one-step RT-RPA-based isothermal method to detect BNYVV from roots—the target tissue of rhizomania in sugarbeet. In addition, for confirmatory checking of the RT-RPA product, optimized conditions for product visualization and downstream sequence analysis revealed the legitimacy of the RT-RPA product, thus validating the method for BNYVV detection. Previously developed molecular methods for BNYVV detection include regular RT-PCR, nested RT-PCR, and IC-RT-LAMP assays (Henry et al., 1995; Morris et al., 2001; Almasi and Almasi, 2017). However, these methods require gel electrophoresis for downstream product analysis, which hinders high-throughput application. In contrast to RPA, LAMP requires four to six primers and consequently tends to generate non-target background amplification due to the involvement of an increased number of primers (Zaghloul and El-Shahat, 2014). In the RT-RPA and CRISPR-Cas system, the first step in the reaction process is sequence-specific target DNA cleavage, which is provided by the guide RNA. After making that cleavage, Cas12 attains a collateral indiscriminate ssDNA cleavage activity, and this feature facilitates generating an enhanced output signal by using an ssDNA reporter and thus increases sensitivity to the assay. Therefore, RT-RPA method is simple, and it offers advantages over other existing molecular-based methods for BNYVV detection since it requires only two primers and can be performed at isothermal temperature with no background and requires no sophisticated instruments.

Rhizomania disease control depends on field soil evaluations that are anticipated to provide informed decisions for varietal selection, an essential strategy for managing the disease. For field soil evaluations, ELISA methods are routinely used because of their ease, high-throughput nature, and commercially available reagents. With the advent of the CRISPR-Cas system, virus diagnostics have been advanced to a new dimension of method developments providing increased sensitivity, specificity, and robustness for high-throughput applications for human viruses (Chen et al., 2018; Curti et al., 2020; Broughton et al., 2020). The CRISPR-based method that we report here can detect BNYVV in the sugarbeet roots baited for rhizomania, generating readily detectable fluorescence signal compared to healthy control root samples. For this assay, we have chosen the BNYVV RNA-1 as the target because the sequence of RNA-1 is among the least divergent component of BNYVV. Consequently, it is less likely to exhibit sequence variations and therefore serves as a reliable target as compared to the other BNYVV RNAs. As part of the method development, we validated the ability of the designed guide RNA at cutting the PCR product to ensure the precision of the assay and determined the sensitivity limit of the CRISPR-based assay using serially diluted template DNA.

Accurate and sensitive virus detection method developments are essential for disease management. The development of CRISPR-Cas-based methods to serve as a diagnostic tool for plant virus detection is emerging, and recently, it has been shown for viruses infecting *N. benthamiana* and apples (Aman et al., 2020; Jiao et al., 2020). Additionally, a CRISPR-Cas method for tomato virus diagnosis was also reported (Alon et al., 2021). The accuracy and specificity of the CRISPR-Cas method in human virus diagnostics (Chen et al., 2018; Curti et al., 2020) support its application as a reliable tool for plant virus diagnosis. The importance of CRISPR-Cas-based diagnostics assays in phytosanitary efforts to produce clean plant materials as part of surveillance programs has been elaborated for virus diagnosis in banana (Tripathi et al., 2021). In this study, we have developed a CRISPR-Cas12a-based method for BNYVV detection in sugarbeet roots applicable for rhizomania diagnosis. We anticipate this approach being of use for the detection of other root-infecting viruses of sugarbeet, including beet soilborne mosaic virus, beet soilborne virus, and beet black scorch virus (Weiland et al., 2020).

In conclusion, we present a CRISPR-Cas-based method for detecting BNYVV in roots of sugarbeet. We first developed a one-step isothermal RT-RPA method for BNYVV detection from rhizomania-infected sugarbeet roots. The RT-RPA method is simple and isothermal as oppose to regular RT-PCR assays. Subsequently, we have developed a CRISPR-Cas12a-based detection method for BNYVV, which has set the stage for sensitive, specific, and high-throughput detection platform for rhizomania evaluation. The development and validation of CRISPR-based BNYVV diagnostic method for sugarbeet roots has advantages in terms of providing sensitivity and robustness under isothermal conditions and, hence, would serve as a valuable tool for sugarbeet industries for evaluating viruses for driving disease management strategies. Moreover, this technology developed for virus diagnostic for underground root tissue can be applied for setting up a CRISPR-based detection platform for other crop-infecting viruses including soil-borne disease-causing agents.

## Data Availability Statement

The original contributions presented in the study are included in the article/supplementary material, further inquiries can be directed to the corresponding author/s.

## Author Contributions

VR: conceptualization, methodology, investigation, data analysis, and writing of the manuscript. JW and MB: rhizomania technical advice, resources, and manuscript revision. JW, MB, and VR: material support. All authors have read and approved the final manuscript for submission.

## Conflict of Interest

The authors declare that the research was conducted in the absence of any commercial or financial relationships that could be construed as a potential conflict of interest.
